# Psychological distress is associated with symptoms of post-traumatic stress disorder among healthcare providers during the COVID-19 pandemic: 2021–2023

**DOI:** 10.3389/fpsyg.2025.1628884

**Published:** 2025-07-17

**Authors:** Brenda L. Coleman, Iris Gutmanis, Robert Maunder, Allison McGeer

**Affiliations:** ^1^School of Public Health, University of Toronto, Toronto, ON, Canada; ^2^Department of Psychiatry, Faculty of Medicine, University of Toronto, Toronto, ON, Canada; ^3^Laboratory Medicine and Pathobiology, Faculty of Medicine, University of Toronto and School of Public Health, University of Toronto, Toronto, ON, Canada

**Keywords:** psychological distress, healthcare provider, COVID-19, post-traumatic stress disorder, Kessler Psychological Distress Scale, Impact of Event Scale-Revised, Canada

## Abstract

**Background:**

During the COVID-19 pandemic, approximately 25% of healthcare providers (HCP) worldwide were reported to have experienced symptoms associated with post-traumatic stress disorder (PTSD). While longitudinal studies have identified factors associated with PTSD in this group of essential workers, associations with psychological distress trajectories have not been studied.

**Methods:**

Healthcare providers who participated in the prospective Canadian COVID-19 Cohort Study were eligible. Baseline data were collected at enrolment with time-varying measures updated by participants every 12 months. Kessler Psychological Distress Scale (K10) questionnaires were completed in March 2021 or upon their recruitment (whichever came first) and every 6 months thereafter. Impact of Event Scale-Revised (IES-R) questionnaires were completed within two weeks of their withdrawal from the study or study termination date (December 2023). Modified Poisson regression was used to assess the association between PTSD symptoms (i.e., IES-R scores of < 24 vs. ≥ 24) and score trajectories of the first four K10 questionnaires that were completed 180 (± 60) days apart.

**Results:**

Of 441 participants, 105 (24.0%) had IES-R scores indicative of concern for PTSD (i.e., ≥ 24). Five trajectories of K10 scores were identified including: resilient (*n* = 111, 25.2%), chronically distressed (131, 29.7%), delayed onset of distress (43, 9.8%), recovery (83, 18.8%), and mutable (73, 16.6%). HCP whose K10 score trajectories were classified as chronically distressed (i.e., all ≥ 16) had rates of IES-R scores indicative of PTSD that were 6.9 times [95% confidence interval (CI) 3.7, 13.0] higher than HCP with resilient score trajectories (i.e., all < 16). Participants with scores in the other three K10 trajectories also had higher rates of IES-R scores of ≥ 24 when compared to those with resilient scores, with adjusted incident rate ratios of 2.6 (delayed onset; CI 1.3, 5.1), 3.1 (recovery; CI 1.4, 7.2), and 4.0 (mutable; CI 2.2, 7.3).

**Conclusion:**

Early and repeated assessment of HCP distress levels will help identify those who are distressed so that evidence-based mitigation strategies can be provided.

## 1 Introduction

Despite the World Health Organization’s declaration of the end of the public health emergency on 11 May 2023 (World Health Organization, 2024), people around the globe continue to get sick and die from severe acute respiratory syndrome coronavirus 2 (SARS-CoV-2). As of 21 September 2024, 60,871 coronavirus disease 2019 (COVID-19)-related deaths had been reported across Canada (Government of Canada, 2022) with another 1,886 deaths reported in the intervening 7 months (Government of Canada, 2025).

Healthcare providers (HCP) continue to provide care to patients with COVID-19, but can come with a long-term emotional toll. Previous literature has shown that public health crises, such as the COVID-19 pandemic, can lead to symptoms of psychological distress (e.g., depression and anxiety) as well as post-traumatic stress disorder (PTSD) in HCP (Naushad et al., 2019). Meta-analyses have estimated that 22%–33% of HCP across the globe experienced depression, 35%–41% reported anxiety, and 21%–32% had symptoms of PTSD during the COVID-19 pandemic (Krishnamoorthy et al., 2020; Boucher et al., 2023). One study found that 20%–23% of Canadian HCP felt anxious, irritable, isolated, or depressed to a “great extent” between April 2020 and February 2022, with the highest estimates for each outcome occurring in March through June 2021 in that study (Boucher et al., 2023).

The 10-item Kessler Psychological Distress Scale (K10) is a 10-question scale to screen for psychological distress that has been shown to discriminate well between cases and non-cases of serious mental illness as defined by the United States Substance Abuse and Mental Health Service Administration (Kessler et al., 2002). It has also been used to assess stress in HCP. Two studies conducted with HCP in Canada during the SARS-CoV-2 pandemic reported rates of distress, as measured by the K10, of 74% in 2020 (Voth et al., 2022) and 70% in 2021 (Gutmanis et al., 2024a).

Post-traumatic stress disorder is a mental health disorder that may occur after someone is exposed to what they perceive as traumatic stress that can lead to chronic impairment and increased risk of co-occurring psychiatric conditions (Mann et al., 2024). PTSD is defined as the presence of symptoms for one or more months; however, symptoms may last for varying lengths of time and may not even become apparent for 6 months or longer (Canadian Psychological Association, 2024). A meta-analysis of HCP with PTSD symptoms following infectious disease outbreaks found that 18.6%–28.4% still had PTSD symptoms after 30 days, 17.7% after 6 months, and 10%–40% after 1–3 years (Preti et al., 2020). A second meta-analysis, focusing on the SARS-CoV-1 pandemic, determined that 16% of HCP reported PTSD symptoms during the pandemic, 19% at 6 months, and 8% more than 12 months afterward (Alberque et al., 2022). A Canadian study that was part of that meta-analysis found that 13–26 months after the SARS-CoV-1 outbreak, HCP who had provided care to patients infected with the virus reported significantly higher levels of post-traumatic stress, as measured with the 15-item Impact of Event Scale (Horowitz et al., 1979), and psychological distress, as measured by the K10, than HCP who did not (Maunder et al., 2006).

Not all HCP respond to the added physical and emotional demands of crises in the same manner. According to Wang et al. (2023), the impact of a traumatic event differs depending on the event itself (intensity and duration) as well as the individual’s perception of the event, personal psychological defense mechanism(s), and support system(s). One systematic review reported that 18%–80% of HCP reported psychological distress during outbreaks caused by caused by SARS-CoV-1, Middle East respiratory syndrome coronavirus, influenza A (H1N1pdm or H7N9), SARS-CoV-2, or the Ebola virus (Preti et al., 2020). A second review assessed responses to potentially traumatic events over time. These authors reported that the most frequently reported trajectories of change in symptoms of PTSD, anxiety, depression, subjective well-being, life satisfaction, psychological functioning, and distress were: (1) resilient, indicating consistently normal scores; (2) chronic, signifying persistently elevated scores; (3) recovery, representing an elevated score immediately following the experience with a return to normal score(s); (4) delayed-onset, indicating initially normal scores followed by elevated score(s); and eight other trajectories that were observed with much lower frequency (Galatzer-Levy et al., 2018). As reported in the review, the same four trajectories were identified among people who had been hospitalized for SARS-CoV-1 in 2003, as measured by the Medical Outcome Study short form health survey (SF-12) (Bonanno et al., 2008). The same four trajectories were reported amongst a representative sample of the Irish adult population during the COVID-19 pandemic (April–December 2020), using a composite score of depression, anxiety, and post-traumatic stress (Hyland et al., 2021).

Canadian HCP participating in the COVID-19 Cohort Study during the pandemic had a rate of distress, as measured by the K10, of 70% in 2021 that dropped to 49% in 2023 (Gutmanis et al., 2024b). In this same study, it was noted that among patient-facing HCP, 48% and 45% of participants had symptoms at levels of concern for PTSD in 2021 and 2022, respectively, with a drop in the rate to 22% in 2023 (Gutmanis et al., 2024a). Although these studies provided rates over time, they failed to describe whether the trajectories of distress were experienced differently within an individual over time. The goal of this explanatory study was to establish the association between psychological distress trajectories as measured by the K10 (Kessler et al., 2002) and PTSD symptoms as measured by the IES-R (Weiss and Marmar, 1997) among Canadian HCP participating in the COVID-19 Cohort Study between March 2021 and December 2023.

## 2 Materials and methods

This analysis is a sub-study of the prospective COVID-19 Cohort Study, a 42 months pan-Canadian (provinces of Ontario, Quebec, Nova Scotia, and Alberta) prospective study to determine the incidence and risk factors for infection with SARS-CoV-2 (Coleman et al., 2024). In short, enrolment occurred from June 2020 to June 2023 with data collection ending upon participant withdrawal or study termination (1 December 2023), whichever occurred first. Prior to recruitment, ethical approval was obtained from each of the 14 participating acute care centers. HCP were eligible for the parent study if they were 18–75 years old at enrolment, provided written consent, were hospital employees who worked ≥ 20 h per week, or were a physician, nurse practitioner, or midwife with hospital privileges or a private practice in Toronto. Participation was voluntary.

For this sub-study, inclusion criteria were COVID-19 Cohort Study participants who submitted their first K10 between 29 March 2021 and 28 March 2022, submitted four K10 surveys with 180 (± 60) days between subsequent submissions, and submitted an IES-R at study withdrawal/completion. Individual observations with incomplete K10 or IES-R scales were excluded from the analysis.

All data were collected anonymously using a bespoke, secure online platform. Baseline data were collected at enrolment with time-varying measures updated by participants every 12 months. All HCP participants were asked to complete a K10 survey in March or April 2021 or upon their recruitment (whichever came first) and every 6 months thereafter. Participants were asked to complete the IES-R within 2 weeks of their withdrawal from the study or at study termination date (1 December 2023).

### 2.1 Explanatory variable (K10 score)

The K10 is a widely-used screening tool of psychological distress (Stolk et al., 2014) that has been used to measure the frequency of symptoms of psychological distress during the COVID-19 pandemic (Gutmanis et al., 2024a). Previous research has established the reliability and validity of the K10 (Andrews and Slade, 2001; Kessler et al., 2003; Brooks et al., 2006; Sampasa-Kanyinga et al., 2018). Items were scored from 1 (none of the time) to 5 (all of the time) with possible scores of 10–50, with higher scores indicating greater distress. These analyses used a score of ≥ 16 to identify those most likely to be experiencing distress (Maunder et al., 2006).

### 2.2 Outcome measure (IES-R score)

The IES-R is a psychometrically sound and widely-used measure of PTSD symptoms (Elhai et al., 2005) that asks participants to indicate how distressing each of 22 difficulties was during the past 7 days on a scale from 0 (not at all) to 4 (extremely) (Weiss and Marmar, 1997). For this study, the IES-R was introduced with “You have been working throughout the COVID-19 pandemic…”. The IES-R cut off scores for this analysis were 0–23 (no concern for PTSD/normal) and ≥ 24 (indicative of concern for PTSD) (Weiss and Marmar, 1997), with three categories of concern including 24–32 (mild), 33–36 (moderate), and ≥ 37 (severe). Subscale scores (avoidance, intrusion, hyperarousal) are the mean of the subscale item scores (range 0–4).

### 2.3 Model covariates

Participant age, gender, and the use of prescription medications for anxiety, depression, or insomnia were collected from the baseline survey completed at enrolment. Other demographic (any children < 19 years of age in the household) and occupational (occupation, working on a high-risk unit, level of patient contact) factors that could vary over time were taken from the baseline survey completed closest, but prior to, the date the fourth K10 was completed. Individuals who indicated that they worked in the emergency department, an adult intensive care unit, or an adult inpatient medical unit were identified as working on a high-risk unit; other work locations were identified as lower risk. The level of contact participants had with each of: inpatients, outpatients, and/or emergency department patients were categorized into the highest level of contact for any of the three settings as (1) no patient care, (2) in patient room, but no patient contact, or (3) physical care/contact. Since nurses have been identified as being more likely to report symptoms of PTSD (Fattori et al., 2023), occupation was dichotomized to nurse (nurse practitioner, midwife, registered nurse/registered practical nurse) or non-nurse (physician, respiratory therapist, laboratory technician, physical therapist, occupational therapist, imaging technician/technologist, pharmacist, pharmacy technician, psychologist, social worker, infection prevention and control practitioner, food service, ward clerk, administration, healthcare aid, housekeeper, porter, researcher, other clinical support).

### 2.4 Data analysis

Change patterns of the dichotomized K10 scores (< 16 vs. ≥ 16) were explored to determine distinct emotional responses using the four previously described trajectories (Galatzer-Levy et al., 2018) as a starting point; they included (1) resilient: all four K10 scores < 16, (2) chronically distressed: all four scores ≥ 16, (3) delayed onset: first score(s) < 16 followed by score(s) always ≥ 16, and (4) recovery: first score(s) ≥ 16 followed by score(s) of < 16. All other trajectories were categorized as mutable since they fit none of the four patterns.

Chi-square, Fisher’s exact tests, or median tests, as appropriate, were used to compare the demographic and occupational characteristics associated with each of the categories. Modified Poisson regression (Zou, 2004) was then used to assess the relationship between the K10 trajectories and IES-R scores (0–23 vs. ≥ 24). Possible demographic and occupational confounding variables were eliminated from a saturated model retaining covariates associated at *p*-values of ≤ 0.2. Covariates that were removed were added back in, one at a time, being retained if the variable changed the adjusted estimate between the trajectory and the IES-R score by ≥ 10%. All variance estimates were adjusted for clustering within province and all models were adjusted for the time between the completion of the fourth K10 survey and the IES-R (per 30 days). Negative binomial models of each of the IES-R subscales that included the variables identified in the previous analysis were then generated using the same procedures as described above. All analyses were done in Stat v.18.1 (StatCorp LLC, 2024).

## 3 Results

**FIGURE 1:**
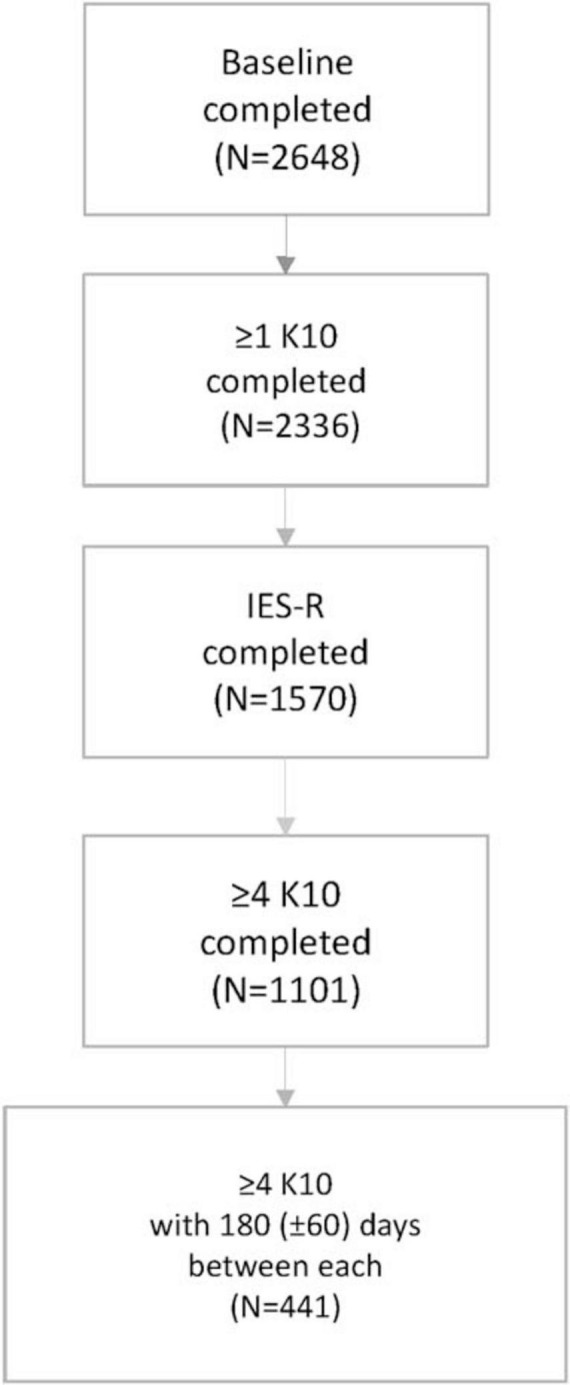
Flow chart of sub-study participation, Canadian healthcare providers, COVID-19 Cohort Study, 4 July 2022–1 December 2023. K10, 10-item Kessler Psychological Distress Scale; IES-R, Impact of Event Scale-Revised.

Of the 2,648 HCP who participated in the parent study, 441 (16.7%) met the sub-study inclusion criteria (see Figure 1). The first K10s were completed between 29 March 2021 and 7 March 2022 while the fourth K10s were completed between 4 July 2022 and 6 November 2023; minimum study participation was 545 days. Most (*n* = 384, 87.1%) IES-R scales were completed in 2023; there was an average of 179 (± 18) days between submission of the fourth K10 and the IES-R. The majority (*n* = 385, 87.3%) of sub-study participants were female, 138 (31.3%) were nurses, 316 (71.1%) reported very good or excellent health, 116 (26.3%) worked on a high-risk unit, and 88 (20.0%) reported using medication to treat anxiety, depression, or insomnia at study enrolment (Table 1).

**TABLE 1:** Characteristics of Canadian healthcare providers who participated in sub-study by K10 trajectory, COVID-19 Cohort Study, 4 July 2022–1 December 2023; number (%) unless otherwise specified.

Characteristic	Study population (*n* = 441)	Resilient^1^ (*n* = 111)	Chronically distressed^2^ (*n* = 131)	Delayed onset^3^ (*n* = 43)	Recovery^4^ (*n* = 83)	Mutable^5^ (*n* = 73)	*P*-value between groups
Age, in yearrs median (IQR)	43 (35, 53)	50 (41, 57)	40 (35, 49)	45 (35, 55)	41 (36, 48)	40 (32, 52)	< 0.001
Gender: female	385 (87.3)	93 (83.8)	122 (93.1)	34 (79.1)	74 (89.2)	62 (84.9)	–
Male	56 (12.7)	18 (16.2)	9 (6.9)	9 (20.9)	9 (10.8)	11 (15.1)	0.07
Subjective health							
Poor/fair/good	125 (28.3)	19 (17.1)	65 (49.6)	9 (20.9)	12 (14.5)	20 (27.4)	–
Very good/excellent	316 (71.7)	92 (82.9)	66 (50.4)	34 (79.1)	71 (85.5)	53 (72.6)	< 0.001
Medicated for anxiety, depression, insomnia	88 (20.0)	11 (9.9)	36 (27.5)	6 (14.0)	18 (21.7)	17 (23.3)	–
Not medicated	353 (80.1)	100 (90.1)	95 (72.5)	37 (86.1)	65 (78.3)	56 (76.7)	0.01
Child in home	144 (32.7)	41 (36.9)	41 (31.3)	9 (20.9)	28 (33.7)	25 (34.3)	–
No child < 18 years	297 (67.4)	70 (63.1)	90 (68.7)	34 (79.1)	55 (66.3)	48 (65.8)	0.43
Household size, median (IQR)	3 (2, 4)	3 (2, 4)	2 (2, 4)	3 (2, 4)	3 (2, 4)	3 (2, 4)	0.62
High-risk unit^6^	116 (26.3)	31 (27.9)	36 (27.5)	9 (20.9)	21 (25.3)	19 (26.0)	–
Lower risk	325 (73.7)	80 (72.1)	95 (72.5)	34 (79.1)	62 (74.7)	54 (74.0)	0.92
Occupation							
Nurse^7^	138 (31.3)	35 (31.5)	32 (24.4)	15 (34.9)	26 (31.3)	30 (41.1)	–
Non-nurse	303 (68.7)	76 (68.5)	99 (75.6)	28 (65.1)	57 (68.7)	43 (58.9)	0.17
Patient contact level							
No patient contact	158 (35.8)	43 (38.7)	46 (35.1)	14 (32.6)	29 (34.9)	26 (35.6)	–
In room, no contact	83 (18.8)	19 (17.1)	26 (19.9)	8 (18.6)	15 (18.1)	15 (20.6)	–
Physical care/contact	200 (45.4)	49 (44.1)	59 (45.0)	21 (48.8)	39 (47.0)	32 (43.8)	1.00
Hours of work, median (IQR)	37.5 (35, 40)	37.5 (32, 40)	37.5 (35, 40)	37.5 (35, 40)	37.5 (35, 40)	37.5 (35, 40)	0.21
K10 scores, median (IQR)	NA^8^	11.8 (10.8, 12.8)	23.8 (20.5, 28.8)	15.3 (14.0, 17.3)	15.8 (14.3, 18.0)	16.0 (15.0, 17.5)	< 0.001
IES-R scores							
0–23 (normal)	335 (76.0)	104 (93.7)	75 (57.2)	36 (83.7)	66 (79.5)	54 (74.0)	–
≥ 24 (of concern)	106 (24.0)	7 (6.3)	56 (42.8)	7 (16.3)	17 (20.5)	19 (26.0)	< 0.001
Year IES-R completed							
2022	57 (12.9)	11 (9.9)	17 (13.0)	5 (11.6)	14 (16.9)	10 (13.7)	–
2023	384 (87.1)	100 (90.1)	114 (87.0)	38 (88.4)	69 (83.1)	63 (86.3)	0.71

^1^Resilient: all four K10 scores were < 16.

^2^Chronically distressed: all four K10 scores were ≥ 16.

^3^Delayed onset: first/first few K10 scores < 16 with all subsequent scores ≥ 16.

^4^Recovery: first/first few K10 scores ≥ 16 with all subsequent scores < 16.

^5^Mutable: K10 scores fell both above and below 16.

^6^High risk units: includes adult intensive care units, emergency departments, and adult inpatient medical units.

^7^Nurse: includes midwives, nurse practitioners, registered nurses, registered practical nurses; Non-nurse HPC: includes administration, food service, healthcare aid, housekeeper, imaging technician/technologist, infection prevention and control practitioner, laboratory technician, occupational therapist, pharmacist, pharmacy technician, physical therapist, physician, porter, psychologist, researcher, respiratory therapist, social worker, ward clerk, other clinical support.

^8^Not applicable because K10 scores changed over time. IES-R, Impact of Event Scale-Revised; IQR, interquartile range; Max, maximum; Min, minimum; NA, not applicable.

The participants in this sub-study are similar to those in the full study. Most were female (85.1%), 33% were nurses, 75.0% reported very good or excellent health, 31.1% worked on a high-risk unit, and 18.1% used medication to treat anxiety, depression, or insomnia. Also, 43.5% [95% confidence interval (CI) 38.5, 48.5] of participants in this sub-study had an average K10 score of ≥ 16 in 2023, which is similar to the rate (49%; CI 44.4, 54.0) reported in a study of the overall K10 scores (Gutmanis et al., 2024a). Similarly, 21.9% (CI 18.0, 26.2) of participants in this sub-study had IES-R scores-of-concern in 2023; this is similar with the 22.5% (CI 18.1, 27.5) reported in 2023 in our study of HCP engaged in patient care (Gutmanis et al., 2024b).

**FIGURE 2:**
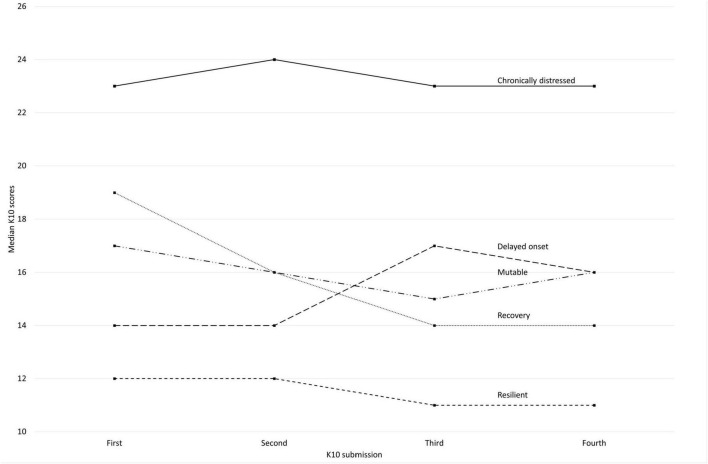
Kessler 10 Psychological Distress Scale median scores by submission (first through fourth) and by response trajectory, Canadian healthcare providers who participated in the COVID-19 Cohort Study, 28 March 2021–1 December 2023. K10, 10-item Kessler Psychological Distress Scale; K10, minimum score is 10; maximum score is 50; scores ≥ 16 indicate possible distress; Trajectories: Resilient: all four scores < 16; Chronically distressed: all four ≥ 16; Delayed onset: first/first few < 16 with all subsequent ≥ 16; Recovery: first/first few ≥ 16 with all subsequent < 16; Mutable: scores were both above and below 16 over time.

Five K10 trajectories of psychological response to working during the COVID-19 pandemic were identified including resilient (*n* = 111, 25.2%), chronically distressed (*n* = 131, 29.7%), delayed onset (*n* = 43, 9.8%), recovery (*n* = 83, 18.8%), and mutable (*n* = 73, 16.6%). As shown in Table 1, the median K10 score varied significantly by trajectory from 11.8 (resilient) to 23.8 (chronically distressed). Figure 2 depicts the mean K10 scores by submission (first, second, etc.) for each trajectory.

### 3.1 Concern for PTSD

The percentage of participants whose IES-R score was > 24 (of concern for PTSD) was significantly higher (22/57 or 38.6%) if submitted between July and December 2022 than if submitted in 2023 (84/384 or 21.9%; *p* = 0.006). The percent of participants with scores > 24 also varied significantly across K10 trajectories, ranging from 6.3% for HCP in the resilient trajectory group to 42.7% in the chronically distressed group (see Table 2).

**TABLE 2:** Median scores for Impact of Event Scale-Revised (overall and subscale) for Canadian healthcare providers, COVID-19 Cohort Study, 4 July 2022–1 December 2023.

Survey scale	Median (IQR)
Total IES-R	10 (2, 23)
Avoidance	0.5 (0, 1.2)
Intrusion	0.5 (0.1, 1.1)
Hyperarousal	0.3 (0, 1.0)

IES-R, Impact of Event Scale-Revised; IQR, interquartile range.

### 3.2 Psychological distress and concern for PTSD

Healthcare providers whose K10 score trajectories were classified as chronically distressed (i.e., all ≥ 16) had rates of IES-R scores indicative of PTSD (i.e., ≥ 24) that were 6.9 times (CI 3.7, 13.0) higher than HCP with resilient score trajectories (i.e., all < 16) after adjusting for occupation and number of days between the submission of the fourth K10 and the IES-R and with variance estimates adjusted for clustering within province (see Table 3). Participants with scores in the other three K10 trajectories also had higher rates of IES-R scores-of-concern for PTSD as compared to those with resilient scores, with adjusted incidence rate ratios (aIRRs) of 2.6 (delayed onset; CI 1.3, 5.1), 3.1 (recovery; CI 1.4, 7.2), and 4.0 (mutable; CI 2.2, 7.3).

**TABLE 3:** Associations between patterns of Kessler 10 scale response trajectories and Impact of Event-Revised scale response groups (< 24 vs. ≥ 24) or sub-scale mean scores, Canadian healthcare providers, COVID-19 Cohort Study, 28 March 2021–1 December 2023; adjusted incident rate ratios^1^ (95% CI).

Scale^1^	Resilient^2^ (*n* = 111)	Chronically distressed^3^ (*n* = 131)	Delayed onset^4^ (*n* = 43)	Recovery^5^ (*n* = 83)	Mutable^6^ (*n* = 73)
Total IES-R (aIRR)	Referent group	6.9 (3.7, 13.0)	2.6 (1.3, 5.1)	3.1 (1.4, 7.2)	4.0 (2.2, 7.3)
Hyperarousal	Referent group	7 (3.4, 6.4)	2.7 (2.0, 3.7)	2.0 (1.1, 3.4)	2.7 (1.9, 3.8)
Intrusion	Referent group	3.2 (2.5, 4.2)	2.1 (1.8, 2.6)	1.9 (1.3, 2.7)	2.2 (1.9, 2.7)
Avoidance	Referent group	2.7 (2.3, 3.3)	1.8 (1.6, 2.1)	1.5 (1.2, 2.0)	1.7 (1.4, 2.1)

^1^Adjusted for months between last K10 and IES-R submission and occupation; variance estimates adjusted for clustering within province.

^2^All four K10 scores < 16.

^3^All four K10 scores ≥ 16.

^4^First/first few K10 scores < 16 with all subsequent scores ≥ 16.

^5^First/first few K10 scores ≥ 16 with all subsequent scores < 16.

^6^K10 scores were both above and below 16 over time. K10, Kessler 10-item scale; IES-R, Impact of Event Scale-Revised.

### 3.3 Subscale (hyperarousal, intrusion, avoidance) models

Subscale Kendel’s tau correlations varied from 0.69 (hyperarousal: intrusion) to 0.61 (hyperarousal: avoidance). As shown in Table 3, the largest difference in the rates of hyperarousal, intrusion, and avoidance were between the resilient group (the referent) and HCPs with scores in the chronically distressed group.

## 4 Discussion

In this longitudinal cohort study of Canadian HCP, five trajectories of psychological responses to working during the COVID-19 pandemic were detected as measured using the K10. These trajectories were associated with higher rates of scores indicative of concern for PTSD (i.e., ≥ 24 as measured with the IES-R). The rate HCPs scoring in the of-concern category of the IES-R was seven times higher for the 30% of HCP whose K10 scores were consistently ≥ 16 (i.e., chronically distressed) than for those who scores were consistently below that cut-off. HCP with K10 scores in other non-resilient trajectories also had higher rates of participants in the of-concern categories. Twenty-four percent of HCP who participated in this study had symptoms at a level indicative of concern for PTSD; 13% had severe symptoms as assessed by the IES-R, demonstrating the considerable long-term emotional health impact of the pandemic among HCP. Given that there are an estimated 2.0 million people working in healthcare in Canada (Government of Canada, 2023), these findings suggest that up to 260,000 Canadian HCP may be in need of psychological assistance following the pandemic.

A temporal relationship between psychological distress and symptoms of PTSD was reported during the first peak of the pandemic in France (Laurent et al., 2022). They found that the odds of having symptoms of PTSD (IES-R scores > 33) were 1.4 times higher for intensive care unit staff who had scores indicative of distress than those with lower scores, as measured 3 months earlier using the General Health Questionnaire. Although the odds of having symptoms of PTSD were lower in the French study than in the current one, there were significant differences in study design including the period and duration of data collection, the measure of distress, and the threshold used for the IES-R.

Authors of a Canadian study of HCP reported that IES-R scores peaked in the spring of 2021 and then decreased through to the spring of 2023 (Maunder et al., 2024). These results are similar to results of our study of Canadian HCP in which 47.8%, 44.8%, and 22.5% had IES-R scores of concern (≥ 24) in 2021, 2022, and 2023, respectively (Gutmanis et al., 2024b). In the current study, participants whose K10 scores indicated a chronically distressed trajectory had the highest rates of IES-R scores indicative of concern for PTSD - and for each of the IES-R’s subscale scores. Authors of a study that followed Canadian HCP ho had survived an infection with SARS-CoV-1 in 2003 reported that levels of depression, anxiety, and PTSD symptoms did not significantly change 1, 4, and 7 years later, demonstrating the long-term impact of pandemics (Moallef et al., 2021). In that study, higher hyperarousal and avoidance scores in 2004 were associated with reductions in functional outcomes. These findings suggest the need to focus prevention and treatment efforts on those in need.

A single assessment can identify HCP who are (or are not) distressed at the time of the measurement. However, as our study highlights, individual levels of distress during a prolonged traumatic event may not be static. In a separate analysis of the K10 data from all HCP in the COVID-19 Cohort Study, we reported that the scores increased during periods of high SARS-CoV-2 transmission but generally decreased between 2021 and 2023 (Gutmanis et al., 2024a). Authors of a second Canadian study of HCP reported that Kessler-6 scores generally mirrored infection rates but failed to decrease in the final year, spring 2022 to spring 2023 (Maunder et al., 2024). In the current study, 25% of participants had K10 score trajectories that represented resiliency while 30% denoted chronic distress. Another 29% had score trajectories that did not remain constant: 10% had delayed onset distress while 19% improved/recovered. In another Canadian study, 53% of HCP who were surveyed for eight consecutive weeks between May 2020 and January 2021 were deemed resilient while 34% exhibited short-term (< 4 weeks) and 13% had longer-term (≥ 4 weeks) distress (Rapisarda et al., 2023). In another study conducted among Canadian intensive care unit staff, of the participants who reported distress at the onset of the pandemic, 45% were persistently distressed while 55% had improved by the end of the first wave of the pandemic (Pestana et al., 2024). Likewise, among Italian HCP with scores above the cut-off for concern in July 2020, 14% were considered chronically distressed, 9% had a delayed onset of symptoms, and 34% had scores suggesting they had recovered by March 2022 (Fattori et al., 2023). The findings are consistent in that levels of distress within many individuals changed over time. As such, it is important to monitor people at regular intervals during a protracted event such as the COVID-19 pandemic.

Assessment should also continue after the end of a prolonged traumatic event since they can have long-lasting impacts on some HCP (Maunder et al., 2006). In that study, more than 40% of HCP who had worked at a hospital that provided care to people hospitalized with SARS-CoV-1 experienced adverse outcomes including burnout, psychological distress, post-traumatic stress, and work-related impacts such as missed work shifts due to stress or illness for more than 18 months after the pandemic had ended. A systematic review and meta-analysis similarly reported that the prevalence of PTSD among HCP was 16% during the 2003 SARS-CoV-1 pandemic and 8% more than one year later (Alberque et al., 2022).

We recognize that there are limitations to this study. Although the questionnaires were completed anonymously, the findings are based on self-reported data that may be subject to over- or under-reporting of symptoms. The data for the K10 and IES-R were collected between February 2021 and December 2023, so results cannot be generalized beyond those dates. The IES-R was only collected once, at the end of the participant’s tenure with the study, so the temporal relationship between PTSD symptoms and trajectories of psychological distress cannot be determined (i.e., the association was not causal). This analysis identifies important vulnerable groups, those with non-resilient K10 trajectories and high PTSD scores long into the pandemic; it does not clarify the relationship between those variables. Also, although participants were from four of the 13 provinces/territories in Canada, the hospitals were located in larger urban centers and participation was voluntary. This limits the generalizability of findings to smaller sub-urban or rural settings, or to HCP at large. Further to this, participation in this sub-study was limited to participants who completed at least four K10 questionnaires. As such, representation of HCP is further limited to those who were able to manage the added workload of participating in the study during the pandemic (i.e., may have been less likely to feel overwhelmed by the added physical and/or emotional burden caused by the pandemic than non-participants) and may not represent all HCP. It is important to keep these limitations in mind when interpreting this study’s results.

This study is one of few that assesses the temporal relationship between symptoms of distress and PTSD and despite the above-mentioned limitations, the study is unique in the duration of data collection (March 2021 to December 2023) and the scope of participation with HCP from 19 hospitals across four Canadian provinces and a variety of occupations. Also, the scales used to measure symptoms of distress and PTSD were validated and are used across the globe.

## 5 Conclusion

Although the majority of HCP did not experience chronic distress during the COVID-19 pandemic, about 40% had chronic or delayed distress as measured over time. This highlights the need to monitor people more than once during a protracted event. Among those with chronic distress, the rate of PTSD symptoms was seven times higher than for HCP who had no symptoms of distress. These people need to be identified and supported. HCP with levels of distress of clinical concern during the COVID-19 pandemic need to be followed over a longer period to understand the multi-year impacts of working during the pandemic.

## CCS Working Group members

Include Kevin Katz, North York General Hospital, Toronto, ON Canada; Louis Valiquette, Centre Hospitalier Universitaire de Sherbrooke, Sherbrooke, QC Canada; Curtis Cooper, University of Ottawa, Ottawa, ON Canada; Robyn Harrison, Alberta Health Services, Edmonton, AB Canada; Mark Loeb, McMaster University, Hamilton, ON Canada; Jeff Powis, Toronto East Health Network, Toronto, ON Canada; Saranya Arnoldo, William Osler Health System, Brampton, ON Canada; Jeya Nadarajah, Oak Valley Health, Markham, ON Canada; Marek Smieja, McMaster University, Hamilton, ON Canada; Samira Mubareka, Sunnybrook Health Science Centre, Toronto, ON Canada; Matthew P. Muller, Unity Health, Toronto, ON Canada; Shelly A McNeil, Dalhousie University, Halifax, NS Canada; and Joanne M Langley, Dalhousie University, Halifax, NS Canada.

## Data Availability

The datasets presented in this study can be found in online repositories. The names of the repository/repositories and accession number(s) can be found below: https://www.maelstrom-research.org/study/cccs.
